# Infections in the era of immunobiologicals^☆^

**DOI:** 10.1016/j.abd.2023.08.004

**Published:** 2024-01-17

**Authors:** Ricardo Romiti, André Luís da Silva Hirayama, Adriana Maria Porro, Heitor de Sá Gonçalves, Luciane Donida Bartoli Miot, Sandra Maria Barbosa Durães, Silvio Alencar Marques

**Affiliations:** a Department of Dermatology, Hospital das Clínicas, Universidade de São Paulo, São Paulo, SP, Brazil; b Department of Dermatology, Escola Paulista de Medicina, Universidade Federal de São Paulo, São Paulo, SP, Brazil; c State Health Secretariat of Ceará, Centro de Dermatologia Dona Libânia, Fortaleza, CE, Brazil; d Department of Infectology, Dermatology, Imaging Diagnosis and Radiotherapy, Faculty of Medicine, Universidade Estadual Paulista, Botucatu, SP, Brazil; e Department of Internal Medicine, Dermatology Unit, Faculty of Medicine, Universidade Federal Fluminense, Niterói, RJ, Brazil

**Keywords:** Biological products, Hepatitis, viral, human, Leprosy, Mycosis fungoides, Tuberculosis, Vaccination, Psoriasis

## Abstract

Immunobiologicals represent an innovative therapeutic option in dermatology. They are indicated in severe and refractory cases of different diseases when there is contraindication, intolerance, or failure of conventional systemic therapy and in cases with significant impairment of patient quality of life. The main immunobiologicals used in dermatology basically include inhibitors of tumor necrosis factor-alpha (anti-TNF), inhibitors of interleukin-12 and -23 (anti-IL12/23), inhibitors of interleukin-17 and its receptor (anti-IL17), inhibitors of interleukin-23 (anti-IL23), rituximab (anti-CD20 antibody), dupilumab (anti-IL4/IL13) and intravenous immunoglobulin. Their immunomodulatory action may be associated with an increase in the risk of infections in the short and long term, and each case must be assessed individually, according to the risk inherent to the drug, the patient general condition, and the need for precautions. This article will discuss the main risks of infection associated with the use of immunobiologicals, addressing the risk in immunocompetent and immunosuppressed patients, vaccination, fungal infections, tuberculosis, leprosy, and viral hepatitis, and how to manage the patient in the most diverse scenarios.

## Introduction

Immunobiologicals (IBs) represent an innovative and effective therapeutic option in medicine and, particularly, in dermatology. They characterize a group of drugs produced by living biological systems through modern biotechnology techniques. They are classified into three main groups: monoclonal antibodies, fusion proteins, and cytokines. They interact with specific human proteins involved in the inflammatory cascade of different immune-mediated diseases, such as psoriasis, atopic dermatitis (AD), hidradenitis suppurativa, urticaria, autoimmune bullous dermatoses and connective tissue diseases.^1^, ^2^, ^3^

With high complexity and structural variability, IBs have a heterogeneous active component that is difficult to characterize and replicate. Due to their complex molecular structure and the large number of steps involved in their development, they characterize high-cost medications with restricted access.^4^

Indicated in severe and refractory cases of different diseases; when there is contraindication, intolerance, or failure of classical systemic therapy; in cases of patients with severe deterioration in the quality of life and/or physical or psychosocial disability, IBs basically include tumor necrosis factor-alpha inhibitors (anti-TNF), interleukin-12 and -23 inhibitors (anti-IL12/23), inhibitors of interleukin-17 and its receptor (anti-IL17), inhibitors of interleukin 23 (anti-IL23), rituximab (anti-CD20 antibody), dupilumab (anti-IL4/IL13) and intravenous immunoglobulin.

For the treatment of psoriasis, which represents the dermatological disease with the highest number of IB approvals, the following can be highlighted, among the drugs with anti-TNF action: infliximab (chimeric monoclonal antibody), etanercept (fusion protein), adalimumab (humanized monoclonal antibody) and certolizumab pegol (pegylated anti-TNF); those with anti-IL12/23 action (ustekinumab), anti-IL23 action (guselkumab, risankizumab), anti-IL17 (secukinumab, ixekizumab, bimekizumab) and anti-IL17 receptor action (brodalumab). Spesolimab (anti-IL36 receptor) was approved in 2023 for the treatment of exacerbations of generalized pustular psoriasis in adults.^5^, ^6^, ^7^, ^8^

IBs do not have specific toxicity for any organ or system; however, their immunomodulatory effect may be associated with the occurrence of short- and long-term adverse effects, such as severe opportunistic infections. Therefore, before introducing specific IB therapy, the therapeutic decision must be shared with the patient and a series of actions and precautions are essential. During treatment, caution must be constant, both on the part of the prescribing physician and the patient. Different specific situations, such as vaccination, surgery and pregnancy, may occur, and it is up to the specialist to discuss the most appropriate course of action with the patient, aiming to reduce the risk of undesirable complications.^9^

Next, the real risk of infections associated with IBs is discussed, both in immunocompetent and immunocompromised patients, the risk of fungal infections and leprosy when taking immunobiological therapy, the appropriate management of cases of co-infection with hepatitis B and C and the importance of adequate vaccination schemes.

## Risk of infection with different immunobiologicals used in dermatology

In patients with immune-mediated diseases treated with IBs, the occurrence of infections may be due to the risk inherent to the underlying disease itself, the immunomodulatory effect of the recommended treatment, the association with immunosuppressive therapies and the presence of different comorbidities, such as obesity and diabetes mellitus.

The high prevalence of skin infections in patients with AD has been well established. Changes in the skin barrier associated with a wide range of immunological changes increase susceptibility to different infections, especially caused by viruses such as herpes simplex, molluscum contagiosum and verruca vulgaris, and by *S. aureus*.^10^ In patients with psoriasis, there are high rates of hospital admission due to infections when compared to the general population, mainly related to the severity of psoriasis and not to the systemic treatments used.^11^ In the case of hidradenitis suppurativa, high rates of cutaneous and extracutaneous infections are observed, possibly resulting from the breakdown of the skin barrier and immune system dysregulation.^12^

The real risk of infection associated with the use of IBs has been evaluated in controlled and randomized studies, in population registries and in systematic reviews of the literature. The indication for psoriasis shows the greatest variety of therapeutic options and a long follow-up period, and the current consensus is that such IBs pose no risk or only minimal risk of infection when compared to classic systemic treatments.^13^ Pharmacovigilance registries developed long term evaluation, such as the British Association of Dermatologists Biologics and Immunomodulators Register (BADBIR) in the United Kingdom, PsoBest in Germany and BIOBADADERM in Spain, have not, to date, shown any warning sign for risk of infections, especially when pre-treatment screening and annual follow-up are carried out appropriately.^1^ On the other hand, Dobry et al., evaluating infection rates in 5,889 patients in the USA, identified an increased risk of severe infections in psoriasis cases using IBs when compared to patients treated with non-IBs (adjusted hazzard ratio [aHR]: 1.31; 95% confidence interval [CI]: 1.02‒1.68), specifically for skin and soft tissue infections (aHR = 1.75; 95%CI 1.19‒2.56).^14^ However, comments subsequent to this publication emphasized the low infection rates in both groups of reported patients (with and without immunobiological treatment) and the relevance of the risk of serious infections inherent to the severity of psoriasis itself, considering that patients with severe disease are more likely to receive treatment with immunobiological drugs.^15^

Real-life data evaluating the risk of severe infections in 11,560 new IB treatments in patients with psoriasis and psoriatic arthritis identified a reduced risk of serious infections in patients treated with anti-IL17 and anti-IL12/23, when compared to the use of anti-TNF in patients without previous exposure to IBs. In bio-experienced patients, there was no difference in the occurrence of serious infections between these three classes.^16^

In general, skin and respiratory infections are the most prevalent when using IBs. On the other hand, one must highlight that the occurrence of granulomatous infections, such as tuberculosis, and fungal infections, mainly by Candida, characterizes a special situation associated with the inherent mechanism of the anti-TNF and anti-IL17 classes, respectively.^6^

Regarding dupilumab, a human monoclonal antibody targeting the α subunit of the IL4 and IL13 receptor and approved for the treatment of moderate to severe AD, the most commonly reported adverse effect is conjunctivitis. Systematic literature reviews and clinical practice meta-analyses show conjunctivitis rates of 26.1% in a total of 908 AD patients. Herpes simplex virus infections were identified in 5.8% of 546 AD cases.^17^

Rituximab features a chimeric anti-CD20 monoclonal antibody that depletes B lymphocytes that have the CD20 surface antigen. It is indicated for a wide variety of diseases, including neoplasms, rheumatological diseases and primary immunodeficiencies, and in some countries (including the United States and Europe), also for pemphigus vulgaris. Off-label indications in Brazilian dermatology clinical practice include pemphigus, systemic lupus erythematosus and angioedema.^18^, ^19^ Among the adverse effects, rituximab is associated with the risk of hypogammaglobulinemia and infections. Although initial studies did not show a significant increase in the risk of infections, reports have shown rates of severe infections of up to 5.2 per 100 patient-years, compared to 3.7 per 100 patient-years in the placebo group, in addition to cases of prolonged and symptomatic hypogammaglobulinemia.^20^, ^21^

## Tuberculosis

**Figure 1:**
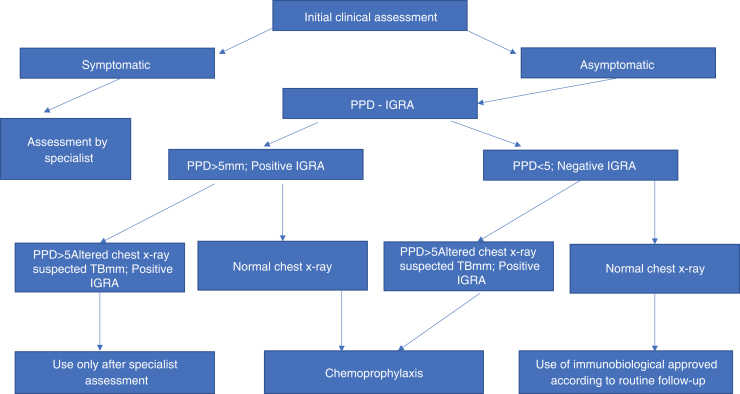
Flowchart of the investigation of latent tuberculosis (TB) in patients candidates for the use of immunobiologicals.

The probability of latent tuberculosis reactivation is greater in patients using anti-TNF. Since the introduction of latent tuberculosis investigation, tuberculosis reactivation rates have decreased dramatically. Therefore, a thorough history regarding current or past contact with tuberculosis is essential. Screening aimed at diagnosing individuals infected with *M. tuberculosis* should include tuberculin skin testing, which uses the standard purified protein derivative (PPD) preparation, and chest radiography (Fig. 1).^22^ Tests carried out *in vitro* for the detection of latent tuberculosis, based on the quantification of IFN-γ production, help with diagnosis, but still have a high cost.^23^ The first versions of these tests (Quantiferon-TB®, Cellestis Limited, Carnegie, Australia), approved by the Food and Drug Administration (FDA) in 2001, used PPD as a stimulating antigen, leading to the same specificity problems observed in skin tests.^24^ Quantiferon-TB-Gold® is an assay using ESAT-6 and CFP-10 as stimulatory antigens. This last assay uses whole blood and quantifies the presence of IFN-γ using the ELISA (enzyme-linked immunosorbent assay) method. The T-SPOT TB® test (Oxford® Immunotec, Marlborough, USA) uses an ELISPOT (enzyme-linked-immunospot) assay using IFN-у-producing peripheral mononuclear cells in response to stimuli such as ESAT-6 and CFP-10.^25^, ^26^

## Use of immunobiologicals in immunocompromised patients

The most important immunocompromised patient populations, in numbers, are patients with HIV (human immunodeficiency virus) and patients undergoing solid organ transplantation.

Although both groups are immunocompromised, they have different types of immunosuppression. HIV infection leads to a progressive impairment of cell immunity, and patients who use ART (antiretroviral therapy) experience restoration of this immunity, with an increase in the serum count of CD4 + T lymphocytes and a decrease in the viral load, which may remain undetectable. When that occurs, patients tend to have longer survival rates and, as a result, can develop autoimmune diseases, inflammatory bowel disease (IBD), and even neoplasms, sometimes generating IB indications.^27^ Solid organ transplantation recipients, in turn, experience more important, complex, and irreversible immunosuppression, as long as the use of immunosuppressive medications persists, which prevents the risk of rejection of the transplanted organ.^28^ It is important to highlight that these two populations are usually excluded from IB clinical studies. Therefore, what is known in this regard is extracted from case reports and series, and few review studies.

### HIV infection and immunobiologicals

HIV infection can trigger and worsen psoriasis, and this probability increases in patients with greater immunosuppression, who have a low count of CD4 + T lymphocytes. Patients with CD4 counts <200 cells/mm^3^ are at increased risk for severe psoriasis.^29^

In a systematic review published in 2019, the authors reviewed cases of psoriasis in special populations, who received treatment with IB between 1989 and 2018. A decrease in CD4 was not observed in patients with HIV infection who received anti-TNF IBs (etanercept, infliximab or adalimumab), nor an increase in the number of opportunistic infections (OIs). The only anti-IL included in this review was ustekinumab (anti-IL12/23), which shows good efficacy and safety profile in this group, including improvement in CD4 count and viral load in some patients.^30^

A multicenter retrospective study assessed 23 patients with HIV infection (five with AIDS) who had moderate to severe psoriasis and used IBs (etanercept, infliximab or ustekinumab) between 2008 and 2016. The mean follow-up was three years: 76% achieved PASI 75, and CD4 was stable or even increased in some patients. One patient who received infliximab developed miliary tuberculosis. The authors concluded that these IBs were effective and had an acceptable safety profile in this population.^31^

In an Italian series of ten patients with HIV infection and psoriasis, published in 2017, six received anti-TNF (four etanercept and two adalimumab), and four received ustekinumab. All achieved PASI 75 within three months. The mean follow-up was 35 months, and none of the patients experienced OIs during this period.^32^

A report of two patients with HIV and erythrodermic psoriasis who received anti-IL17 (one secukinumab and the other ixekizumab) showed they had rapid control of the erythroderma, without infectious complications.^33^

There are also reports of the use of anti-IL23 to treat psoriasis in patients with HIV infection, demonstrating its efficacy and safety.^34^

A recently published systematic review on the use of dupilumab in patients with HIV infection assessed 27 cases (23 published and four from the authors personal experience). Of these, 96% showed improvement in asthma or AD, without changes in the viral load in 100% of them and no changes in CD4 in 80%. The authors concluded this medication is safe if used in patients with stable CD4 counts and a low viral load.^35^

### Solid organ transplantation and immunobiologicals

A systematic review published in 2021 evaluated the safety of using IBs in solid organ transplantation recipients. A total of 111 articles were reviewed, 57 of which were case series, with a total of 187 patients. Of these, 141 had received liver transplants, 42 kidney transplants, three heart transplants, and one kidney-liver transplant. Regarding the immunosuppressive medications used by these patients, the most frequently used were calcineurin inhibitors, followed by corticosteroids, sodium mycophenolate and, finally, mTOR inhibitors. Regarding the IB indication, 81% of patients had IBD, 7.5% had rheumatological indications, 6% had hereditary periodic fever and psoriasis was present in 5%. The most frequently used IB class was anti-TNF (78% of patients). It was found that 29% of the patients had infections, most frequently bacterial and viral infections by cytomegalovirus and herpes simplex. Neoplasms occurred in 9% of the patients, mainly colorectal tumors and non-melanoma skin cancer. There were nine deaths among the 187 patients, but the authors conclude that it is difficult to establish a direct relationship between the use of IBs and death, due to the large number of variables involved.^28^

Regarding anti-TNF, several articles mention an increased risk of non-melanoma skin cancer and severe infections, also as a cause of death, in solid organ recipients.^36^, ^37^

As for anti-IL, the number of publications is small, especially because they are IBs with more recent use. There are published reports on the use of anti-IL12/23 (ustekinumab) for psoriasis and IBD with good response to treatment and no complications.^38^, ^39^ Regarding anti-IL17, there are two reports of patients with psoriasis who received ixekizumab, with good psoriasis control and without complications.^40^

The psoriasis treatment guideline of the American Academy of Dermatology mentions that IBs (anti-TNF, anti-IL12/23, anti-IL17 and anti-IL23) can be used in patients with HIV infection, provided they are receiving ART, have normal CD4, undetectable viral load and do not have recent OIs. There is no mention of the use of IBs in solid organ transplantation recipients.^41^

The Brazilian Consensus on Psoriasis 2020 also suggests that IBs can be used in patients with HIV infection, assessed on a case-by-case basis, and that they would possibly be safer for these patients than immunosuppressants such as methotrexate and cyclosporine. There is no mention of their use in transplanted patients in this Consensus, either.^6^

In conclusion, regarding the use of IBs in immunocompromised patients, studies suggest that, in patients with HIV infection, among the IBs, anti-IL can be preferably used only in those receiving regular ART therapy, with undetectable viral load, normal CD4 count, and no recent infections. IB use should be avoided in solid organ transplant recipients, but when absolutely necessary, anti-ILs seem to be safer than anti-TNFs.

## Fungal infections and immunobiological therapy

The real risk of fungal infections secondary to IB use is difficult to determine due to the inherent predisposition to fungal infections in patients with autoimmune diseases, the concomitant use of immunosuppressants and the geographic endemicity of some mycoses.^42^

### Anti-TNF

As early as in the first decade of their use, some reviews drew attention to the association of fungal infections with anti-TNF drugs,^43^, ^44^, and in 2008 the FDA warned about the increased risk of invasive fungal infections after 240 cases of histoplasmosis.^45^ Two decades later, there is some evidence of OIs occurring with the use of these drugs.^42^, ^43^ Anti-TNF drugs have been associated with a small but significant increase in the risk of severe infections. Adalimumab and infliximab are associated with a higher risk of OIs, including fungal infections such as histoplasmosis, coccidioidomycosis, candidiasis, and aspergillosis, than etanercept.^46^ However, after a larger number of patients have been treated over time, it can be considered that fungal infections are infrequent. In a study of more than 30,000 patients treated with anti-TNF during 2007–2009, 158 patients (0.51%) developed mycoses or mycobacterioses; about half of these infections were fungal, including histoplasmosis, pneumocystosis, cryptococcosis, coccidioidomycosis, and blastomycosis.^47^ Regarding the new anti-TNFs, a recent study found that the risk of severe infection with certolizumab was not significantly higher than with other anti-TNFs.^48^ The safety of golimumab was verified in a recent large series in which infections were the most common adverse effect; however, no fungal infections were observed.^49^

### Anti-IL

Psoriatic patients using ustekinumab had a lower frequency of severe infections than those observed during treatment with infliximab and other IBs. A long-term safety assessment included 1,482 patients with at least four years of exposure to ustekinumab and no fungal infections were reported.^50^

**Figure 2:**
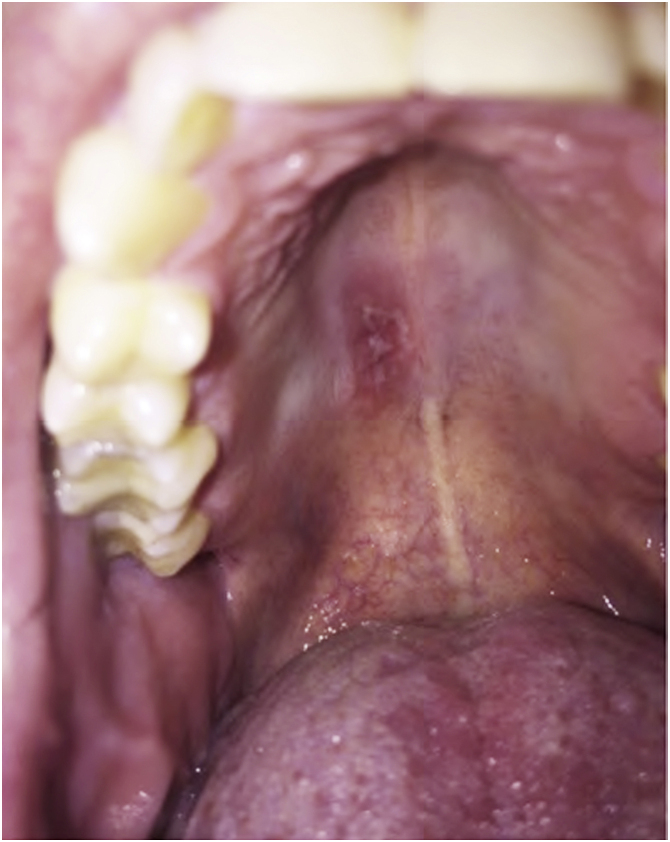
Oral candidiasis with the use of anti-IL17.

Primary immunodeficiencies that result from mutations in the genes encoding IL17 increase susceptibility to Candida infections. As expected, in patients who received anti-IL17 (brodalumab, secukinumab and ixekizumab) there was an increased incidence of candidiasis. However, most of the time the therapeutic response was good, without the need to withdraw the anti-IL17 treatment.^51^ Fig. 2 depicts a case of oral candidiasis while using anti-IL17.

The risk of systemic fungal infections with anti-IL23 treatment in patients with psoriasis was investigated in 16 randomized controlled trials. No cases of severe fungal infections were observed in patients treated with risankizumab, guselkumab, and tildrakizumab.^52^

A meta-analysis including eight controlled trials showed that patients treated with dupilumab had a lower risk of skin infection and a non-significant increase in herpes infections. The normalization of the skin barrier, microbiome, and treatment of the aberrant immune response in AD are possible explanations.^53^

### Rituximab

The risk of invasive fungal infections following treatment with anti-CD20 monoclonal antibodies seems to be low and pneumocystosis could be an exception, as some studies have suggested a possible increased risk.^54^ However, current evidence supports that anti-pneumocystis prophylaxis should be considered only in those also receiving other concomitant immunosuppressive drugs.^55^

Autoimmune diseases already carry an inherent risk of infections and therefore it is important to assess the specific risk of infection from immunosuppressive medications in each disease. Despite some variability, in systemic immune-mediated diseases, there is a greater probability of using multiple immunosuppressants and in higher doses, differently from what occurs in dermatological patients. The concomitant use of other immunosuppressants is a well-established factor in increasing the risk of fungal infections in patients taking anti-TNF drugs.^56^

A review of 19 studies to evaluate the incidence of fungal infections in patients taking IBs for psoriasis included 4,547 patients taking etanercept, infliximab, adalimumab, or ustekinumab. There was one case of coccidioidomycosis in a patient using adalimumab, and no severe fungal infections were reported.^57^

A recent retrospective study verified the benefit of prophylaxis with co-trimoxazole for the risk of *Pneumocystis* pneumonia (PCP) in patients with pemphigus using rituximab. The medical records of 494 patients were reviewed, of which 235 received co-trimoxazole, whereas 259 did not. The incidence of PCP was low, with no significant difference between the groups (p = 0.84). The authors concluded that there is no consensus on the need for prophylaxis due to the low incidence of PCP complications in these patients, and it is not mandatory to treat all pemphigus patients with rituximab.^58^

### Endemic fungal infections in Brazil

Paracoccidioidomycosis (PCM) is a systemic mycosis caused by *Paracoccidioides brasiliensis* and *Paracoccidioides lutzii*, endemic and limited to Latin America, from Mexico to Argentina. Although uncommon, PCM has been reported in patients with immunosuppression, with the majority of these cases occurring in patients with HIV. Only three cases of IB-related PCM have been reported.^59^, ^60^, ^61^ In these patients, the infection appeared more than a year after the introduction of the medication.

In recent decades, sporotrichosis has emerged as an endemic mycosis associated with feline transmission in southern and southeastern Brazil. As with PCM, disseminated sporotrichosis has been described in patients with AIDS, and there are few reports of the disease in patients with other immunosuppressive conditions and few with biological therapy.^62^ As the use of IBs is increasing, these cases, although rare, demonstrate the importance of including PCM and sporotrichosis in the OI list in patients from endemic areas on long-term immunotherapy.^63^

## Leprosy and immunobiologicals

In 2004, data from the FDA Adverse Event Reporting System (FAERS) were analyzed to verify the incidence of granulomatous infections during anti-TNF use. Up to that date, 346,000 patients had been treated with infliximab and etanercept, and 639 infectious adverse events had occurred. In the infliximab group, there was only one case of leprosy, but no additional information was provided about the patient.^43^

A Brazilian multicenter study assessed data from 1,037 patients (750 treated with IBs and 287 controls). Infections occurred in 23% of patients using IBs, most of them in the upper airways, three cases of tuberculosis and only one case of tuberculoid leprosy.^64^

In 2020, a systematic review analyzed the incidence of leprosy in patients using anti-TNF. In the end, ten patients developed leprosy in Brazil (n = 6), the United States (n = 2), Greece (n = 1) and Spain (n = 1). Leprosy is endemic in Brazil and India and the latter reports the highest number of leprosy cases globally, with 114,451 cases in 2019; thus, the authors state that patients from India should be on this list. They considered that financial difficulties and lack of availability could reduce the use of IB products in endemic countries and explain the absence of leprosy cases in India.^65^

A recent cohort study evaluated the risk of leprosy in patients using IBs and conventional immunosuppressants for dermatological and rheumatological diseases. A total of 405 patients were included (268 immunosuppressed) from 2014 to 2019. Ten cases of leprosy were diagnosed (nine in immunosuppressed patients). Corticosteroid use has been associated with an increased risk of leprosy, and anti-TNF use has been associated with a lower risk of leprosy when compared to corticosteroids.^66^

### Treatment of leprosy reactions with immunobiologicals

Leprosy reactions are acute immunoinflammatory events that occur in some patients in response to *Mycobacterium leprae* antigens. They are often accompanied by neuritis and increase the chance of disability. The small number of published cases, nine in total, indicates that anti-TNF drugs may be useful in treating individuals with chronic refractory reactions or those unable to tolerate standard therapies due to adverse effects. Since even chronic reactional states tend to progress with decreasing severity and frequency over time, randomized clinical trials are needed to establish the role of anti-TNF drugs in the treatment of severe reactions.^65^, ^67^

## Hepatites and immunobiologicals

The exact risk of hepatitis B and C reactivation in patients undergoing biological therapy is not defined due to the fact that these patients cannot participate in pivotal studies of the medications, due to exclusion criteria.

It is estimated that hepatitis B may affect more than 370 million people worldwide. The prevalence of hepatitis B in psoriasis in the United States is 0.5% and of hepatitis C is 1.3%, and there is no prevalence data available in Brazil to date in this population.^6^, ^68^, ^69^ In patients with pemphigus, studies estimate that the prevalence of hepatitis B is 0.6%‒1.2%.^70^, ^71^

All patients must undergo serology tests for hepatitis B and hepatitis C before starting immunobiological therapy.

### Hepatitis B

It is recommended that before starting immunobiological therapy, all patients undergo serology for hepatitis B, including Anti-HBc (antibody against the core of the hepatitis B virus), AgHBs (surface antigen of the hepatitis B virus) and Anti-HBs (antibody against the surface antigen of the hepatitis B virus). Table 1 shows possible serological profiles and their interpretations.^69^, ^70^, ^71^

**Table 1:** Interpretation of hepatitis B serological profiles.

**Profile 1**	Anti-HBs	Negative	Susceptible
	Anti-HBc	Negative	
	AgHBs	Negative	
**Profile 2**	Anti-HBs	Positive	Previous exposure
	Anti-HBc	Positive	
	AgHBs	Negative	
**Profile 3**	Anti-HBs	Positive	Immunized by the vaccine
	Anti-HBc	Negative	
	AgHBs	Negative	
**Profile 4**	Anti-HBs	Negative	Acute infection
	Anti-HBc	Positive	
	Anti-HBc IgM	Positive	
	AgHBs	Positive	
**Profile 5**	Anti-HBs	Negative	Chronic infection
	Anti-HBc	Positive	
	Anti-HBc IgM	Negative	
	AgHBs	Positive	
**Profile 6**	Anti-HBs	Negative	4 possible scenarios^a^
	Anti HBc	Positive	
	AgHBs	Negative	

a Possible scenarios: 1) Patient recovering from acute hepatitis B; 2) Patient may have a previous infection and the test might not be sensitive enough to detect very low levels of anti-HBs; 3) False positive for anti-HBc; 4) Possible undetectable level of HBsAg, and patient is chronically infected – possible occult hepatitis B (anti-HBs can be positive in these cases too).

All susceptible patients should be vaccinated against hepatitis B, whenever possible, before starting immunobiological treatment.

All patients with a history of hepatitis B must have their viral DNA measured before starting IB treatment; therefore, AgHbs-positive patients must have their viral DNA tested. The test should also be considered in the case of negative AgHBs with positive anti-HBc, in an attempt to detect occult hepatitis B.^6^, ^72^, ^73^, ^74^, ^75^

The risk of hepatitis B reactivation seems to be higher in patients with chronic hepatitis B infection and lower in patients with previous infection or occult hepatitis.^6^, ^72^, ^73^

## Use of chemoprophylaxis

The use of chemoprophylaxis is recommended for patients with chronic hepatitis B and should be considered in patients with previous infections and occult hepatitis B who are undergoing immunosuppression. Some authors consider that prophylaxis should only be used in patients with cancer undergoing chemotherapy and solid organ transplant patients.^76^, ^77^

It must be started before treatment and continued for six to 18 months after its end. The drugs of choice are tenofovir and entecavir.^78^

**Figure 3:**
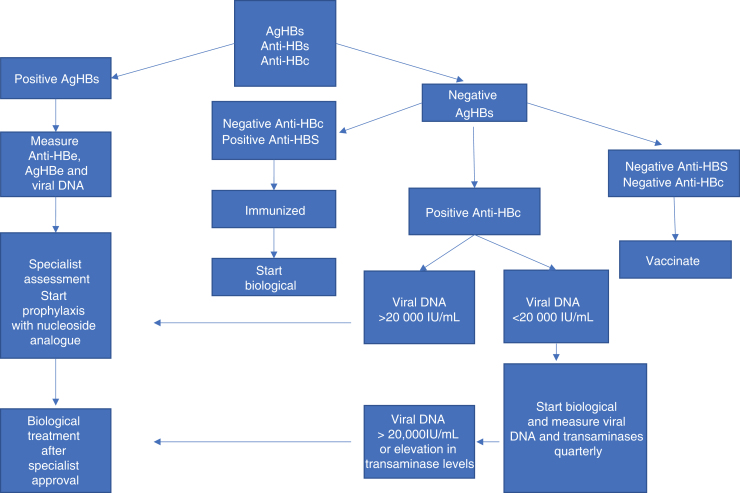
Conduct in cases of immunobiological use and hepatitis B. Adapted source: Romiti R, et al. 2020.^6^

Fig. 3 summarizes the conduct in cases of IB use and hepatitis B.

## Hepatitis B and the use of IBs

The use of TNF-alpha antagonists seems to be associated with higher rates of hepatitis B reactivation when compared to ustekinumab and secukinumab.^72^, ^73^

There are studies, however, that place ustekinumab at a moderate risk for hepatitis B reactivation, which may be associated with IL12 blockade, which would play an important role in controlling viral replication.^78^, ^79^

Some authors suggest that, in patients with psoriasis and chronic or previous hepatitis B infection, the first choice should be the use of anti-IL17, followed by anti-IL23; however, the evidence to support this recommendation is scarce and more studies are needed to support it.^72^

### Hepatitis B and dupilumab

The safety of dupilumab in patients with hepatitis B is uncertain. Pivotal dupilumab studies excluded patients with hepatitis B.^80^

Case reports suggest there is no increased risk of hepatitis B reactivation with the use of dupilumab.^81^

Despite little evidence regarding patients with chronic or previous hepatitis B infection, some authors suggest that in cases of severe AD in which the use of systemic therapy is indicated, dupilumab should be the drug of choice, before other immunosuppressants.^82^

## Hepatitis B and rituximab

Data on hepatitis B reactivation in patients with pemphigus using rituximab are scarce. Studies suggest that the risk of hepatitis B reactivation in these patients is not as high as in patients with hematological malignancies or solid organ transplant recipients using rituximab. The use of rituximab in the treatment of patients with pemphigus seems to be safer than the use of high doses of corticosteroids, causing lower rates of hepatitis B reactivation.^71^

In these cases, it is suggested that antiviral prophylaxis be started before the use of IBs and maintained during treatment for up to 18 months after its end, with monitoring of viral DNA and transaminases.^71^

### Hepatitis C and the use of IBs

Regarding hepatitis C and psoriasis, the use of IBs is contraindicated during acute hepatitis.^69^, ^83^, ^84^, ^85^, ^86^

The risk of hepatitis C reactivation in patients with chronic hepatitis C and low levels of viral RNA seems to be very low.^69^, ^83^, ^84^, ^85^, ^86^

IBs can be used with caution, under the supervision of a specialist. Measurements of transaminases and viral RNA must be performed quarterly.

Previous studies have even demonstrated that the use of anti-TNF led to reductions in the patients viral load. Anti-IL17 drugs seem to have a favorable safety profile in this population. The safety of anti-IL23 drugs in patients with psoriasis and chronic hepatitis C remains uncertain.^69^, ^83^, ^84^, ^85^, ^86^

To date, there is no clear evidence that the use of rituximab worsens the course of hepatitis C in patients with pemphigus. The risk-benefit of using the medication must be discussed with the specialist (gastroenterologist; hepatologist; infectious disease specialist) and compared to other available therapies to provide the best option for each case.^87^

There is scarce data in the literature on the use of dupilumab in patients with hepatitis C, and the safety profile remains uncertain. However, some authors recommend the use of dupilumab as the first-choice therapy in patients with severe AD who require the use of immunosuppressants to control the disease.^82^

## Vaccination: how to proceed in patients undergoing immunobiological therapy

The risk of infections is greater in patients with chronic immune-mediated diseases, such as psoriasis, AD and pemphigus vulgaris. Moreover, there is a higher rate of certain types of infections in patients on IB therapy, particularly respiratory infections, followed by herpes zoster.^88^, ^89^

The greater occurrence of herpes zoster in the elderly and immunocompromised individuals generates significant morbidity, which can result in pain, depression, post-herpetic neuralgia, encephalitis, an ophthalmological disease with loss of vision and neurological manifestations such as Ramsay-Hunt syndrome. In immunocompromised patients, ophthalmological involvement occurs in up to 25% of patients and post-herpetic neuralgia in up to 30% of them.^90^

Reactivation of hepatitis B with IB treatments is low. Therefore, vaccination is one of the preventive measures with the greatest impact on reducing the occurrence of hepatitis, its complications and sequelae in any age group.^6^

Due to greater susceptibility to infections, it is suggested that updating the vaccination record of patients who have indications for IB therapy be implemented, aiming to reduce the risk of some infections and, consequently, reduce the morbidity and mortality rate in this specific group of patients.^91^ Preferably, the vaccination card should be updated two to four weeks before starting potentially immunosuppressive therapies, including IBs.^92^, ^93^ Vaccination of contacts is also an important guideline.

Live attenuated vaccines are generally contraindicated in patients undergoing treatment with immunomodulators; however, the risk for complications depends on the degree of immune suppression and individual risk factors. Inactivated agent vaccines can be applied during the use of systemic immunomodulatory therapies, although there is a risk of reducing their immunogenicity.^93^ Table 2 depicts a list of the main vaccines and the characteristics of the IB agents (inactivated or attenuated).^6^

**Table 2:** Main vaccines and characteristics of the biological agents.

Inactivated agents	Attenuated agents
Pentavalent (diphtheria, tetanus, pertussis, *Haemophilus influenzae* and polio)	MMR (measles, mumps and rubella)
Hexavalent (pentavalent and Hepatitis B)	Tetra viral (MMR + varicella)
Pneumococcal	Varicella
Inactivated polio vaccine (IPV)	Yellow Fever
Trivalent or quadrivalent influenza	Oral Polio Vaccine
Adult Td (tetanus and diphtheria)	BCG (if mother is taking anti-TNF wait six months for baby vaccination)
HPV	Rotavirus
*Haemophilus influenza* B	Herpes Zoster (Zostavax®)
Hepatitis B	Dengue (Qdenga®)
Hepatitis A	
COVID-19 (including recombinant mRNA)	
Pneumococcal conjugate vaccine 13	
Pneumo-23 Vaccine	
Meningitis ACWY (MenACWY) vaccine	
Meningitis B vaccine	
Herpes Zoster (Shingrix®)	

During treatment with IBs, it is necessary to evaluate the epidemiological situation, the medication being used and disease activity, aiming to choose the best time for vaccination.^92^, ^93^ The search for windows of opportunity for vaccination must be carried out. Disease control *versus* the risk of drug discontinuation must always be assessed on an individual basis. Whenever possible, the vaccination schedule must be completed, taking into account the intervals between the withdrawal of the drugs being used and immunization.^91^, ^92^, ^93^, ^94^

Regarding the period of IB application withdrawal before and after the use of vaccines with live or attenuated agents, it is recommended to suspend treatment at least four to five half-lives before the vaccination and wait approximately 30 days after vaccination for the reintroduction.^95^

Patients should be advised regarding the possibility of receiving passive immunization at Reference Centers for Special Immunobiologicals (CRIE, *Centros de Referência para Imunobiológicos Especiais*) considering exposure to specific situations, when this resource is available: human immunoglobulin for anti-hepatitis B, anti-rabies, anti-varicella, and anti-tetanus vaccines.

Flu vaccination should be recommended for all patients. For those with low complement levels or functional asplenia, vaccines against *Haemophilus influenza* type B, *pneumococcus* and *meningococcus* should be indicated. The pneumococcal conjugate vaccine 13 (PCV13) and herpes zoster vaccine should be considered for all adults (over 19 years of age) on immunosuppression or IB therapy.^92^, ^93^

More recently, a non-live recombinant vaccine was developed to prevent herpes zoster (Shingrix®), which is the only one recommended in immunocompromised patients. The immunization consists of two intramuscular doses, the first in month zero and the second two to six months afterwards.^96^

Another important issue is that the necessary immunization with live attenuated components should only be carried out after the reading of the PPD test, as both should not be performed simultaneously, due to the risk of a false-negative result. If the patient has received any of these vaccines, PPD should only be carried out after four weeks.^97^

Patients who do not know how to provide information about their vaccination history or who have lost their immunization card should have the card redone with all vaccines indicated for their age group, considering themselves as not previously vaccinated.

Available serologies should be requested to check whether the patient is susceptible to any of the vaccine-preventable diseases or to confirm the response after vaccine administration. For varicella, all patients who deny previous illness or cannot remember it should be considered susceptible.^98^

It is recommended to assess seroconversion for hepatitis B in patients who were immunized while on immunosuppressive therapy, and before starting the immunobiological. Serological tests to assess seroconversion, when possible, should be performed, and additional doses of immunizers should be administered if this has not been adequately carried out.^99^

It is worth noting that the use of anti-TNF agents (mainly certolizumab and infliximab), male gender and vaccination for hepatitis B after starting treatment with anti-TNF are risk factors for non-response to this vaccine. Therefore, the hepatitis B vaccine must be administered before starting anti-TNF treatment or within six months after starting it.^100^ Regarding the new option of the pneumococcal conjugate vaccine for adults (PCV13), assessing the serological response is not recommended, as this is not representative of the immunological status.^101^

Another important point is that adverse event rates are similar between children who were exposed *in utero* to anti-TNF agents before the third trimester of pregnancy and those who were exposed in the third trimester. All adverse events spontaneously resolved and no severe events occurred, such as tuberculosis activation or death. BCG administration after six months of age brings low risk to children exposed to anti-TNF agents *in utero*. However, BCG vaccination should not be delayed beyond 12 months, even when drug testing is not possible. Even in children who received the BCG vaccine within the first six months after birth, the risk was low and showed no severe adverse events. The cost-benefit of whether or not to carry out the vaccination early must be decided on an individual basis and analyzed according to the epidemiology of each location.^102^

In relation to COVID-19, none of the vaccines for population use in Brazil are live attenuated viruses and, therefore, can be used by patients using immunobiological medications, unless there is any specific contraindication.

The effectiveness of vaccines may be reduced due to the use of immunosuppressive medications; however, to date, there are no studies on the effects of vaccines in patients with chronic diseases and/or using IBs.^103^

Regarding monkeypox, there are three possible vaccines: ACAM2000, LC16 and MVA-BN (Jynneos/Imvanex). LC16 is a minimally replicating immunizer, being the most modern and most frequently used. This is the immunizer approved by ANVISA and purchased by the Brazilian government. As it is a low-replicating attenuated virus vaccine, it can be used in immunocompromised individuals.^104^

## Conduct guideline

Below is a guideline on how to evaluate and monitor patients using IBs, both before and during treatment.

### Pretreatment

**Figure 4:**
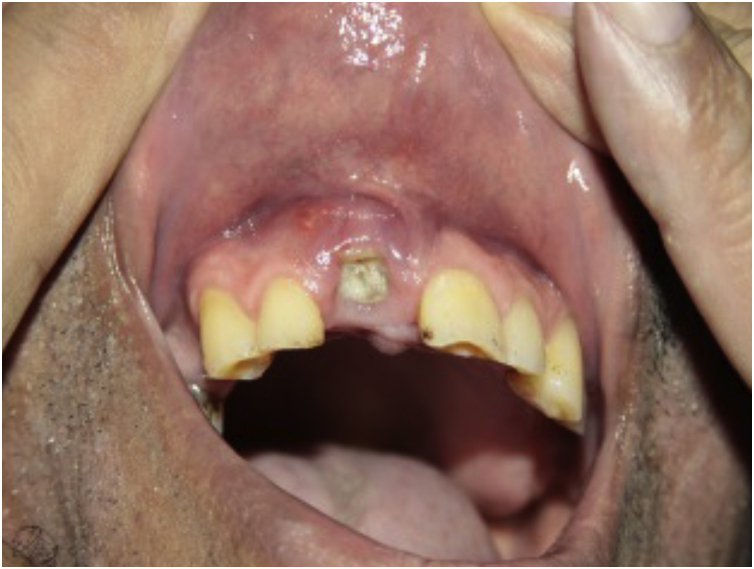
Dental abscess before starting immunobiological.

Perform a complete history and physical examination. Check the epidemiological history, especially the place of origin and recent travel. In the anamnesis and during the clinical examination, be alert for signs and symptoms of infection (exemplified in Fig. 4), neoplasms (including skin cancer), pregnancy, demyelinating disease, and other autoimmune diseases, as well as liver and cardiovascular disease. In patients with psoriasis, evaluate the musculoskeletal system to investigate psoriatic arthritis, dactylitis, or enthesitis.^26^, ^105^

Actively looking for signs of infection, before and during biological therapy, should include^26^, ^105^ pulse and temperature; oroscopy and otoscopy; lymph node palpation; auscultation of the heart and pleuropulmonary fields; abdomen palpation; inspection of the entire integument, including nails and mucous membranes; dental health inspection.

General guidelines: treatment with IBs should not be started in the presence of infections, including chronic or localized infections, and is completely contraindicated in patients with septicemia or at risk for septicemia. The patient should be warned to avoid contact with people with severe and contagious infections and seek medical evaluation when any sign or symptom of infection is present. Studies have shown that the risk of severe infections is greater during the first six months of treatment with anti-TNF IBs, mainly when compared to subsequent years.^16^

The vaccination calendar must be updated. The risk of the presence of comorbidities must be discussed with the patient and their identification and follow-up must be part of the investigation routine. Therefore, measurement of blood pressure levels, weight and pelvic girdle measurements should be recorded before and during treatment. 26, 105

Below are the contraindications to the introduction of IBs for patients with psoriasis:^106^

Absolute contraindications: severe acute infection (e.g., active tuberculosis); hypersensitivity to any of the active ingredients or excipients; Need to receive live attenuated virus vaccines concomitantly with treatment;

Relative contraindications: chronic infections (hepatitis B or C, HIV); malignancies or lymphoproliferative disorders.

Laboratory tests must be requested before and during IB therapy. Specific investigation of autoimmune diseases should be requested in the presence of signs of these diseases during the anamnesis and/or physical examination.^26^, ^106^, ^107^ Table 3 depicts the tests to be requested as well as the frequency of their performance.

**Table 3:** Exams for screening and during immunobiological therapy follow-up.

Exam	PRE	Every 2 to 5 months
Complete Blood Count	X	X
Liver Enzymes	X	X
Renal function	X	X
Urinalysis	X	X
Pregnancy test	X	X
PPD or IGRA and chest X-ray	X	When indicated
Hepatitis and HIV serologies	X	When indicated

### Follow-up

Careful history taking and physical examination must be carried out periodically during IB therapy. Active and continuous surveillance for tuberculosis in patients treated mainly with anti-TNF drugs is mandatory, especially in countries with high rates of the disease or in individuals living in endemic areas.^26^, ^106^, ^107^ Routine tests should preferably be requested every two to five months during follow-up (Table 3).

## Financial support

None declared.

## Authors' contributions

Ricardo Romiti: Design and planning of the study; Data collection, or analysis and interpretation of data; drafting and editing of the manuscript or critical review of important intellectual content; collection, analysis, and interpretation of data; critical review of the literature; approval of the final version of the manuscript.

André Luís da Silva Hirayama: Design and planning of the study; data collection, or analysis and interpretation of data; drafting and editing of the manuscript or critical review of important intellectual content; collection, analysis, and interpretation of data; critical review of the literature; approval of the final version of the manuscript.

Adriana Maria Porro: Design and planning of the study; data collection, or analysis and interpretation of data; drafting and editing of the manuscript or critical review of important intellectual content; collection, analysis, and interpretation of data; critical review of the literature; approval of the final version of the manuscript.

Heitor de Sá Gonçalves: Design and planning of the study; data collection, or analysis and interpretation of data; drafting and editing of the manuscript or critical review of important intellectual content; collection, analysis, and interpretation of data; critical review of the literature; approval of the final version of the manuscript.

Luciane Donida Bartoli Miot: Design and planning of the study; data collection, or analysis and interpretation of data; drafting and editing of the manuscript or critical review of important intellectual content; collection, analysis, and interpretation of data; critical review of the literature; approval of the final version of the manuscript.

Sandra Maria Barbosa Durães: Design and planning of the study; data collection, or analysis and interpretation of data; drafting and editing of the manuscript or critical review of important intellectual content; collection, analysis, and interpretation of data; critical review of the literature; approval of the final version of the manuscript.

Silvio Alencar Marques: Design and planning of the study; data collection, or analysis and interpretation of data; drafting and editing of the manuscript or critical review of important intellectual content; collection, analysis, and interpretation of data; critical review of the literature; approval of the final version of the manuscript.

## Conflict of interest

Ricardo Romiti: Abbvie - advisory board, consultant, speaker, researcher; Boehringer-Ingelheim - advisory board, consultant, speaker, researcher; Elli-Lilly - advisory board, consultant, speaker, researcher; Janssen - advisory board, consultant, speaker; Leo Pharma - advisory board, consultant, speaker; Novartis - advisory board, consultant, speaker; UCB-Biopharma - advisory board, consultant, speaker.

André Luís da Silva Hirayama: Abbvie - speaker, researcher; Boehringer-Ingelheim - consultant, speaker, researcher; Eli-Lilly - researcher; Novartis - consultant, speaker.

Adriana Maria Porro: Abbvie - advisory board; Boehringer-Ingelheim - advisory board; Janssen - advisory board, researcher; Leo Pharma - speaker; Novartis - advisory board; Pfizer - Other.

Heitor de Sá Gonçalves: None.

Luciane Donida Bartoli Miot: Abbvie - speaker, researcher; Janssen - speaker; Novartis - speaker.

Sandra Maria Barbosa Durães: None.

Sílvio Alencar Marques: Abbvie - researcher; Boehringer-Ingelheim - consultant.
